# The Anticancer Potential of Plant-Derived Nutraceuticals via the Modulation of Gene Expression

**DOI:** 10.3390/plants11192524

**Published:** 2022-09-26

**Authors:** Maria Vrânceanu, Damiano Galimberti, Roxana Banc, Ovidiu Dragoş, Anamaria Cozma-Petruţ, Simona-Codruţa Hegheş, Oliviu Voştinaru, Magdalena Cuciureanu, Carmina Mariana Stroia, Doina Miere, Lorena Filip

**Affiliations:** 1 Department of Toxicology, “Iuliu Haţieganu” University of Medicine and Pharmacy, 6 Pasteur Street, 400349 Cluj-Napoca, Romania; 2 Italian Association of Anti-Ageing Physicians, Via Monte Cristallo, 1, 20159 Milan, Italy; 3 Department of Bromatology, Hygiene, Nutrition, “Iuliu Haţieganu” University of Medicine and Pharmacy, 6 Pasteur Street, 400349 Cluj-Napoca, Romania; 4 Department of Kinetotheraphy and Special Motricity, “1 Decembrie 1918” University of Alba Iulia, 510009 Alba Iulia, Romania; 5 Department of Drug Analysis, “Iuliu Haţieganu” University of Medicine and Pharmacy, 6 Pasteur Street, 400349 Cluj-Napoca, Romania; 6 Department of Pharmacology, Physiology and Physiopathology, “Iuliu Haţieganu” University of Medicine and Pharmacy, 6 Pasteur Street, 400349 Cluj-Napoca, Romania; 7 Department of Pharmacology, University of Medicine and Pharmacy “Grigore T. Popa” Iasi, 16 Universităţii Street, 700115 Iași, Romania; 8 Department of Pharmacy, Oradea University, 1 Universităţii Street, 410087 Oradea, Romania

**Keywords:** nutraceuticals, gene expression, epigenetic therapy, cancer

## Abstract

Current studies show that approximately one-third of all cancer-related deaths are linked to diet and several cancer forms are preventable with balanced nutrition, due to dietary compounds being able to reverse epigenetic abnormalities. An appropriate diet in cancer patients can lead to changes in gene expression and enhance the efficacy of therapy. It has been demonstrated that nutraceuticals can act as powerful antioxidants at the cellular level as well as anticarcinogenic agents. This review is focused on the best studies on worldwide-available plant-derived nutraceuticals: curcumin, resveratrol, sulforaphane, indole-3-carbinol, quercetin, astaxanthin, epigallocatechin-3-gallate, and lycopene. These compounds have an enhanced effect on epigenetic changes such as histone modification via HDAC (histone deacetylase), HAT (histone acetyltransferase) inhibition, DNMT (DNA methyltransferase) inhibition, and non-coding RNA expression. All of these nutraceuticals are reported to positively modulate the epigenome, reducing cancer incidence. Furthermore, the current review addresses the issue of the low bioavailability of nutraceuticals and how to overcome the drawbacks related to their oral administration. Understanding the mechanisms by which nutraceuticals influence gene expression will allow their incorporation into an “epigenetic diet” that could be further capitalized on in the therapy of cancer.

## 1. Introduction

Nowadays, cancer is the second leading cause of death globally, with nearly one in seven deaths being due to cancer. About one-third of deaths from cancer are determined by five main behavioral and dietary risks: high Body Mass Index (BMI), low fruit and vegetable intake, lack of physical activity, tobacco use, and alcohol consumption [1]. Several clinical and epidemiological studies support the association between nutrition and the development or progression of different malignancies [2], with prostate, colon, gastric, and breast cancer being the types of cancer most closely related to diet. There is scientific evidence that a proper diet and lifestyle can substantially reduce cancer risk. For example, adherence to the Mediterranean diet (MD) has been reported as a valuable tool against cancer, and several studies have found a significant reduction in cancer mortality in subjects following the MD. A diet designed to support cancer patients can help reduce the toxicity of radio- and chemotherapy and strengthen the immune system. In the last years, science has focused more on nutraceuticals as protective factors [3]. According to the scientific literature, nutraceuticals are a good source of molecules, able to regulate gene expression and reverse epigenetic alterations due to specific modulation mechanisms [4]. Nutraceuticals, therefore, assume the role of cellular and functional modulators, able to ensure the optimization of the physiological processes of the human body [5].

In the field of cancer research, epigenetic modifications are of particular interest, having an impact on cell proliferation, differentiation, and survival [6]. Cancer can be considered a multi-stage heterogeneous disease, driven by genetic and epigenetic anomalies. Epigenetic changes are involved in biological diversity, aging, and the pathogenesis of cancer and other diseases. All human cancers are characterized by epigenetic changes that cooperate with genetic alterations [7], allowing the uncontrollable growth of cells. The epigenetic transformations are represented by post-translational changes in nucleosomal histones, the most common being methylation, acetylation correlated with transcriptional activation, deacetylation correlated with transcriptional repression, and DNA methylation and regulation by non-coding RNAs [8,9]. The epigenetic component is influenced by exogenous and endogenous factors, including diet, lifestyle, environment, ethnicity, drug intake, exposure to toxins, xenobiotics, age, sex, exercise, and family genetic heritage. Epigenetic therapy is a new area for the development of nutraceuticals, whose low risk of toxicity can represent a valid asset in the cancer prevention strategy [10]. The great potential of this type of therapy lies in the fact that epigenetic alterations are reversible, aiming to reprogram cells to a normal state [11].

Recent advances in understanding nutrigenetics and nutrigenomic mechanisms have led to the identification of nutraceuticals and biocompounds capable of favorably influencing gene expression. A healthy diet and a balanced lifestyle combined with targeted and personalized integration can keep people healthier, favoring successful aging and preventing diseases. Nutraceuticals are able to provide the elements necessary to supply the body’s defense store and to optimize the responsiveness of whole organs, intervening in DNA repair processes and counteracting the key factors correlated with whole-body aging and disease progression. Bioavailability, metabolism, and the tissue distribution of bioactive molecules derived from nutraceuticals are key factors that must be managed accurately in association with their biological effects, not only in vitro but also in vivo [12].

## 2. Methodology

We conducted a narrative literature review, using the academic databases Pubmed and ScienceDirect for the search and collection of literature. Major keywords, such as “nutraceuticals”, “cancer”, “gene expression”, “microRNAs”, “bioavailability”, “bioactive compounds”, “curcumin”, “resveratrol”, “sulforaphane”, “indole-3-carbinol”, “astaxanthin”, “quercetin”, “epigallocatechin-3-gallate”, “lycopene”, and “in vitro”, “in vivo”, and “clinical studies”, were used individually or in combination during the literature survey. We considered original research articles written in English and based our search on their importance and relevance to the field. Due to the large number of published articles on nutraceuticals included in the study, as well as the limited number of references allowed, it was necessary to focus on the most impactful and relevant aspects, and we included published review articles where appropriate. In general, we focused on recently published articles but did not impose limits on the date of publication.

## 3. Nutraceuticals

The term nutraceutical combines the words nutrition and pharmaceutical and indicates those nutrient principles that are found within foods. These have beneficial health effects. Nutraceutical substances derive mainly from plants, food, and microbial sources. This term was invented in 1989 by Dr. Stephen L. De Felice, who, by combining the words “nutrition” and “pharmaceutical”, came to the term “nutraceutical” to indicate a food substance that, for its functional properties, aligns precisely with the limit between food and drug [13]. In reality, we should distinguish between nutraceuticals and functional foods, where the first indicates a specific substance extracted from food, with certain medicinal qualities, while the second means a real (or added) food that directly shows beneficial properties through its introduction into a diet. Nutraceuticals are biological substances that are considered as foods, parts of foods, or dietary supplements with preventive, rebalancing, therapeutic, and protective properties. Recent studies have shown promising results for these compounds in numerous pathological complications such as cancer, diabetes, and cardiovascular or neurological disorders [14]. All these conditions are characterized by many changes, including alterations in the redox state, and most nutraceuticals have antioxidant activity with the ability to fight against this situation [15].

These natural molecules are often plant extracts titrated for a particular active ingredient. In the group of nutraceuticals, there are several substances, among which the best known are curcumin, resveratrol, astaxanthin, sulforaphane, indole-3-carbinol, quercetin, epigallocatechin-3-gallate (EGCG), lycopene, anthocyanins, ellagic acid, fisetin, capsaicin and extracts of ginger (*Zingiber officinale* Rosc.), *Ziziphus jujuba* Mill., *Uncaria tomentosa* Willd. ex Schult., *Silybum marianum* L., and *Bacopa monnieri* L., all covering various therapeutic areas and having the ability, according to the latest studies, to modulate gene expression [16,17,18].

For each of the plant-derived nutraceuticals selected in this study, Table 1 summarizes the plant source, their ability to modulate gene expression and regulate microRNAs, and their antitumor effect.

**Figure 1:**
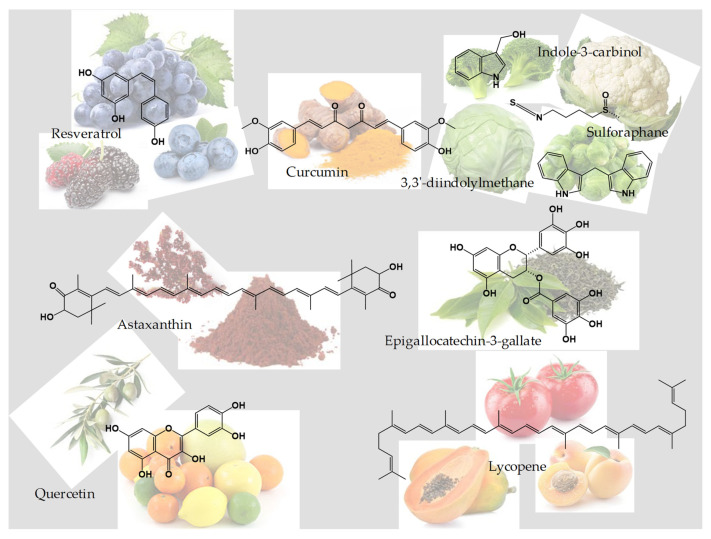
Main plant-derived nutraceuticals.

In this review, we have focused on the first eight of those previously mentioned as the most well-known nutraceuticals, also illustrated in Figure 1, because these are the most studied in terms of antioxidant and anticancer properties, as well as the most targeted to be used in the treatment of cancer as adjuvants in association with chemotherapeutic drugs such as gemcitabine, docetaxel, doxorubicin, and cisplatin, to enhance their efficiency or limit their toxicity. Indeed, in the international database of clinical studies (ClinicalTrials.gov), it can be observed that curcumin, resveratrol, sulforaphane, indole-3-carbinol, quercetin, EGCG, and lycopene are currently in clinical trials on various types of cancer. Several examples of such clinical studies can be seen in Table 2.

### 3.1. Curcumin

*Curcuma longa* L. is an herbaceous plant, perennial and rhizomatous, which belongs to the family of Zingiberaceae, as ginger (*Zingiber officinale* Rosc.) also does. The root, which is the most important component of phytotherapeutic and nutritional interest, is constituted by a cylindrical, branched, aromatic rhizome of orange-yellow color. It is used in food as a spice, especially in traditional Indian, Middle Eastern, and Thai cuisine. The plant contains more than 100 chemical compounds, but the term curcumin generally refers to 1,7-bis(4-hydroxy-3-methoxyphenyl)-1,6-heptadiene-3,5-dione, a compound known as “curcumin I”. Two other best-known compounds are curcumin II (demethoxycurcumin, 1-(4-hydroxy-3-methoxyphenyl)-7-(4-hydroxyphenyl)-1,6-heptadiene-3,5-dione) and curcumin III (bisdemethoxycurcumin, 1,7-bis(4-hydroxyphenyl)-1,6-heptadiene-3,5-dione) [77]. The specific and well-known yellow curcumin color is due to “curcumin I” and the curcuminoids, bisdemethoxycurcumin and demethoxycurcumin, generally used as a natural dye in the food industry [78]. The principal essential oils of curcumin are turmerone (ar-turmerone), β-turmerone, α-turmerone, β-bisabolene, β-sesquiphellandrene, α-zingiberene, curcumol, and curcumenol [79].

Curcumin is famous for its antioxidant, anti-inflammatory, and anticancer properties and recently has been shown to act as an epigenetic modulator [25]. The role of curcumin as an epigenetic regulator includes histone modification by the regulation of histone acetyltransferase (HAT) and histone deacetylase (HDAC); DNA methylation by the inhibition of DNA methyltransferase (DNMT); microRNA modulation by the upregulation of tumor-suppressive miRNAs (miR-15a, miR-16, miR-22, miR-26a, miR-34a, miR-145, miR-146a, miR-200b, c, miR-203, and let-7) [19,20]; the downregulation of oncogenic miRNAs (miR-19a, b, miR-21, miR-27a, miR-130a, miR-186) [24]; and the activation of transcription factors, cytokines, and tumor suppressor genes [26]. DNA methylation is a great target in the treatment of acute myeloid leukemia (AML) as it is well known that the inactivation of genes due to DNA methylation has a major role in the development of AML. It has been shown that curcumin is able to downregulate DNMT1 expression in AML cell lines, in vitro and in vivo [27]. p65 and Sp1 expression, positive regulators of DNMT1, may be reduced by curcumin, which correlates with reductions in the binding of these transcription factors to the DNMT1 promoter in AML cell lines. These characteristics of curcumin make it a promising compound in the treatment of AML [28]. Due to the changes in DNA methylation, curcumin is a hypomethylating agent in breast, prostate, colon, and lung cancer.

Curcumin is able to target other, different cancer-related pathways, such as tumor suppressor genes, growth-signaling factors, transcription factors, apoptotic genes, oncoproteins, the biomarkers of inflammation, or protein kinases [29].

#### 3.1.1. Anticancer Activity and the Suppression of Carcinogenesis

One of the main mechanisms of the anticancer effects of curcumin is due to its interference in the cell cycle and reduction in cyclin-dependent kinase (CDK) expression that controls cell-cycle progression [80]. Curcumin is able to suppress the human epidermal growth factor receptor 2, a tyrosine kinase (HER2-TK), and in this manner inhibits breast cancer cell lines [81]. By administering curcumin, there is a decrease in the activation of the PI3K (phosphoinositide 3-kinase)/AKT (AKT serine/threonine kinase) signaling pathway, resulting in an anticancer effect via the negative modulation of this cell-signaling pathway [30].

Curcumin can modulate the activity of different transcription factors, inhibiting some of them, such as nuclear factor-κB (NF-κB), activated protein-1 (AP-1), signal transducer and activator of transcription (STAT) proteins, hypoxia-inducible factor-1 (HIF-1), Notch-1, early growth response-1 (Egr-1), and β-catenin, but activating others, such as NF-E2-related factor (Nrf2) [29,31]. Transcription factors play an important role in various stages of carcinogenesis, being involved in cell proliferation, cell survival, invasion, angiogenesis, and inflammation. Most of these factors are upregulated in most cancers [31]. It has been demonstrated that curcumin inhibits STAT3 phosphorylation, which is responsible for signaling carcinogenic pathways [21]. Furthermore, curcumin is a potent inhibitor of NF-κB, and this effect is correlated with cellular apoptotic response [22]. Likewise, curcumin stimulates the expression of pro-apoptotic Bax and inhibits the activation of Mcl-1 and Bcl-2 (apoptosis regulator) antiapoptotic agents, also altering the expression of apoptotic mechanisms associated with NF-κB proteins, p38 and p53 [23].

#### 3.1.2. Inhibition of Angiogenesis

In some tumors, curcumin inhibits angiogenesis by suppressing angiogenic cytokines, such as *IL*-6, *IL*-23, and *IL*-1*β* [82], and it is a direct inhibitor of angiogenesis by downregulating transcription factors, such as NF-κB, and proangiogenesis factors, such as bFGF (basic fibroblast growth factor), VEGF (vascular endothelial growth factor), and MMPs (matrix metalloproteinases), all of them linked with tumorigenesis [83].

#### 3.1.3. Anti-Inflammatory Properties

Curcumin is a highly pleiotropic molecule, able to interact with numerous molecular targets involved in the inflammatory process, hence the strong anti-inflammatory action both in the acute phase and in the chronic phase of inflammation. Due to its strong anti-inflammatory effects, in several studies, curcumin showed the ability to prevent the development of some types of cancer by reducing the production of COX-2, lipoxygenase 2, iNOS, and related cytokines, known as mediators of the inflammatory process [84].

Furthermore, curcuminoids are able to exert antioxidant action by blocking free-circulating radicals and inhibiting the formation of new ones [85]. Curcumin can also increase the antioxidant activity, in vitro and in vivo, of the enzymes SOD, CAT, GST, and GSR, and, in this manner, curcumin directly inhibits the formation of reactive species, including superoxide radicals, nitric oxide radicals, and hydrogen peroxide. On the other hand, curcumin also increases the activity of detoxifying enzymes by reducing xenobiotics, therefore protecting against carcinogenic processes [86]. In light of these facts, research is aimed at clarifying the beneficial effects of the combination of curcumin with various antineoplastic drugs so as to improve their clinical effects and reduce their toxicity [81,87].

Although curcumin has significant medicinal properties, its poor bioavailability has limited the success of in vivo epigenetic studies, only partly bypassed by the possibility of using high dosages of the active ingredient in relation to its very low toxicity. Recently, pharmaceutical research has led to the introduction in the market of molecules with better bioavailability (phytosome technology), also opening new therapeutic horizons in terms of preventive medicine and antiaging [88].

The extremely poor bioavailability of curcumin is due to its low aqueous solubility, poor absorption, and rapid metabolism and elimination [77,89,90]. Curcumin is a hydrophobic polyphenol, practically insoluble in water between pH 1–6 [90,91]. Although some studies indicate the dissolution of curcumin in slightly basic water or aqueous buffer, there is, however, no extraordinary increase in solubility under more alkaline conditions. Moreover, curcumin becomes very susceptible to degradation, particularly around neutral pH, i.e., at a pH above 6.5 [89,92].

The low absorption rate of curcumin in the gastrointestinal tract is due to the hydrophobic nature of curcumin [77]. A series of clinical studies analyzed by Nelson et al. showed that although curcumin was administered in a high oral dose of up to 12 g/day, which was well tolerated, the absorption of the compound was negligible and curcumin could not be detected in the serum of most subjects tested [93]. Dei Cas and Ghidoni confirmed, in other two studies performed on healthy volunteers, that curcumin was detected only in the plasma of one of the subjects from the first study, and only in the plasma of two of the twenty-four subjects enrolled in the second study, respectively, and only after a high single oral dose of 10–12 g [91]. Regarding the distribution of curcumin through the body, one study shows that the compound is degraded and/or transformed before and/or after absorption, while the results of several studies suggest that curcumin is not distributed to any specific organ at appreciable levels [93]. The liver is the main organ responsible for the metabolism of curcumin, along with the intestine and intestinal microbiota. In humans, phase I metabolism consists of the reduction of the double bonds of curcumin, in enterocytes and hepatocytes, through the action of alcohol dehydrogenase, forming mostly dihydrocurcumin, tetrahydrocurcumin, hexahydrocurcumin, and octahydrocurcumin, while dihydroferulic acid and ferulic acid are minor biliary metabolites [77,90,91,93]. Curcumin and its reduced metabolites are then subjected to phase II metabolism by conjugation with glucuronic acid and sulfate at the phenolic positions [94]. Intestinal microbiota, through *Escherichia coli* and *Blautia* sp., has been shown to be responsible for an alternative metabolism of curcumin. Several studies suggest that some of the curcumin metabolites may be more active than curcumin [90,91]. After oral administration, curcumin and its metabolites are mostly excreted in the feces, the urinary excretion being extremely low [77,94].

Moreover, the clinical use of curcumin may be also limited by its photodegradation in light, being affected both the structure and properties of curcumin [95].

In order to overcome the main disadvantages related to the oral administration of curcumin, new strategies for its efficient delivery have been investigated. Among the curcumin formulation strategies used in order to enhance its absorption are lipid additions (such as turmeric oil, piperine, or turmeric oleoresin), the adsorption and dispersion of curcumin onto various matrices (such as γ-cyclodextrin or whey protein), and particle size reduction, but also modified structures of curcumin analogs and micellar and nanoparticle formulations of curcumin [94,96]. Unfortunately, some of these formulations claimed an enhanced bioavailability of curcumin only on the basis of increased solubility, without considering the solubility–permeability interplay in the gastrointestinal tract when using solubility-enabling formulations for oral lipophilic drugs [94,97]. The most important goals in the development of curcumin delivery systems are enhancing solubility, increasing bioavailability by enhancing small intestine permeation, preventing degradation in the intestinal environment, increasing content in the bloodstream, and increasing efficacy. Among the delivery systems that have shown promising results in this regard are micelles, liposomes, phospholipid complexes, nanoemulsions, microemulsions, emulsions, solid lipid nanoparticles, nanostructured lipid carriers, biopolymer nanoparticles, microgels, nanogels, etc. [94,96,98,99,100].

### 3.2. Resveratrol

Resveratrol (3,5,4′-trihydroxystilbene) is a stilbenoid, a polyphenolic phytoalexin produced by some plants in response to injury or attack by pathogens, such as fungi or bacteria. Sources of resveratrol in food include grapes (*Vitis vinifera* L.), blueberries (*Vaccinium corymbosum* L.), raspberries (*Rubus idaeus* L.), mulberries (*Morus alba* Hort. ex Loudon L.), and peanuts (*Arachis hypogaea* L.). Resveratrol presents two geometric isomers: *cis-*(*Z*) and *trans-*(*E*). The *trans* form exposed to ultraviolet radiation can undergo isomerization to the *cis* *form* [101]. The *cis* form is dominant in prevalence and especially in= biological activity such as cell-cycle arrest, apoptosis, differentiation, and the anti-proliferation of cancer cells [102,103]. Originally, resveratrol was isolated by Takaoka in 1940, from the roots of white hellebore (*Veratrum album* L.), and in 1963, from knotweed (*Polygonum cuspidatum* Sieb. et Zucc) root. However, only in 1992 did resveratrol attract attention when its presence in wine was associated with the cardioprotective effects of this beverage. *Polygonum cuspidatum* Sieb. et Zucc. is one of the richest sources of resveratrol in nature and, for this reason, it has become a very important plant in modern herbal medicine [104,105].

#### 3.2.1. Antioxidant and Anti-Inflammatory Activity

Resveratrol is able to exert powerful antioxidant and anti-inflammatory action. As an antioxidant, it has a superior activity to that of more known molecules, such as vitamin C and E, and is also more effective than flavonoids because it also acts upstream of the reaction, rendering copper inactive as a catalyst through its chelation [106].

In addition to the direct antioxidant effect, resveratrol also regulates the gene expression of the prooxidant and antioxidant enzymes: SOD1 and GPX1 are strengthened by resveratrol in a concentration-dependent manner. Therefore, the suppression of the expression of the prooxidant genes (via NADPH-oxidase) and the induction of antioxidant enzymes, such as SOD1 and GPX1, are important components of the antioxidant protective effect induced by resveratrol [107]. Resveratrol has been proven to be an effective scavenger of free radicals, including superoxide radical (O^2−^), hydrogen peroxide (H_2_O_2_), hydroxyl radical (OH^−^), nitric oxide (NO), and nitrogen dioxide (NO_2_) [108,109].

However, direct scavenger activities are relatively scarce, also due to the reduced in vivo half-life of this molecule. The antioxidant properties of resveratrol in vivo, on the other hand, are due to its effect as a regulator of gene expression. Resveratrol induces the downregulation of NADPH-oxidase, with a consequent reduction in reactive oxygen species (ROS). Furthermore, by hyperstimulating tetrahydrobiopterin-GTP-cyclohydrolase, the expression of a variety of antioxidant enzymes is increased. Some of the genes regulating the effect of resveratrol are mediated by Nrf2 [110].

#### 3.2.2. Resveratrol and Cells Apoptosis

The role of resveratrol as a modulator of cell apoptosis is fundamental. The cellular apoptosis promoted by resveratrol can be mediated by multiple mechanisms, such as the upregulation of cyclin-dependent kinase inhibitors; the activation of mitochondria and cascade of caspases, apoptosis-inducing cytokines, and related receptors; the downregulation of cell survival proteins (e.g., survivin, XIAP (X-linked inhibitor of apoptosis protein), cIAPs, cFLIP, Bcl-XL, Bcl-2); and the inhibition of cell survival kinases (e.g., MAPK, AKT/phosphoinositide 3-kinase (PI3K), PKC, EGFR kinase) and transcription survival factors (e.g., NF-κB, AP-1, HIF-1α, and signal transducer and transcription activator (STAT3)). The induction of one of these pathways by resveratrol leads to cell death [32,33].

Resveratrol regulates proteins involved in DNA and cell-cycle synthesis, such as p53 and Rb/E2F, CDK, and their inhibitors. Resveratrol influences the activity of transcription factors involved in proliferation and stress response, such as NF-κB, AP1, and EGR1. One part of these events is mediated by MAPK and tyrosine kinase, for example, SRC, and leads to the modulation of survival and apoptotic factors (e.g., members of the Bcl-2 family, inhibitors of apoptosis) as well as to the modulation of enzymes involved in carcinogenesis (e.g., cyclooxygenase (COX), nitric oxide synthase (NOS), phase I and II enzymes) [32]. Finally, resveratrol helps regulate the activity and expression of co-transcription factors such as p300 and SIRT1 [34].

A limited number of studies have demonstrated that resveratrol administration leads to the restoration of the hyper- and hypomethylated states of several oncogenic and tumor suppressor genes [111]. In HCC1806 breast cancer cells, resveratrol downregulates the DNMT1, DNMT3a, DNMT3b, and negatively regulated hTERT with the inhibition of SIRT followed by the inhibition of breast cancer cell growth [112]. In colon cancer, the administration of resveratrol increases SIRT1 expression and decreases NF-κB, with antiproliferative effects on colon cancer cell lines. In prostate cancer cells, resveratrol downregulates the metastasis-associated protein 1, MTA1, allowing the acetylation/activation of p53 [113]. Resveratrol is able to inhibit cell proliferation and metastasis by modulating genes involved in cell cycle regulation and by upregulating p53, leading to enhanced apoptosis [35]. Regarding microRNA modulation, resveratrol decreases several oncogenic microRNAs, including miR17, miR25, and miR 92a-2, and increases the expression of tumor-suppressive miR 34a and miR 663 in colon cancer [36,37]. In breast cancer, resveratrol increases the expression of miR-141 and miR-200 with the inhibition of the proliferation of cancer cells [38]. In light of these studies, resveratrol appears capable of exerting antioxidant and chemopreventive activities and could be considered an epigenetic drug.

**Figure 2:**
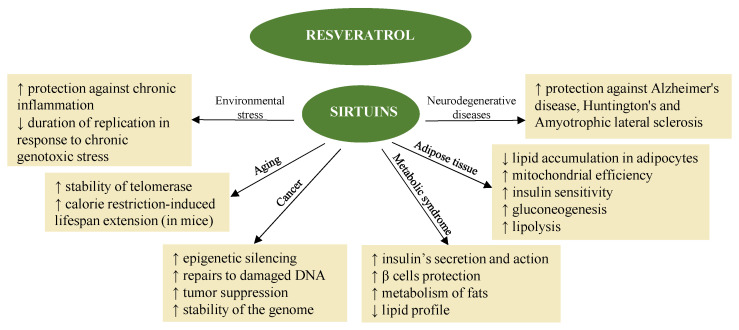
Resveratrol exerts different effects by activating sirtuin [4].

Resveratrol also promotes the activation of sirtuins [39] (Figure 2) in synergy with melatonin. The fact that melatonin and resveratrol are present in various foods implies possible synergistic effects, suggesting combined use to promote health and longevity [114].

The mechanism of epigenetic action in the case of resveratrol also suggests its indication in the treatment of neurodegenerative diseases [115,116]. In fact, the neuroprotective and neurotrophic effects induced by resveratrol have been the subject of multiple studies, both in vitro and in vivo, and make it again a dietary epidrug in the adjuvant treatment and prevention of these diseases [116]. Resveratrol induces autophagy, directly inhibiting the mTOR pathway through interaction with the ATP-binding pocket of mTOR (it direct competes with ATP) [117]. Likewise, it induces the death of tumor cells, also thanks to the inhibition of the mTORC1 pathway [118].

It is necessary to remember that the TOR gene and the expressed mTOR protein are modulated by nutrients and regulate cell growth, motility, proliferation, survival, and protein synthesis and transcription, acting as kind of centralized modulators of various metabolic signals [118,119].

However, the reasons that limit the effectiveness of resveratrol in vivo are dosage and bioavailability. The bioavailability of resveratrol is very low because of its very fast metabolism [70]. Despite having high cellular membrane permeability and being a lipid-soluble compound, resveratrol has poor water solubility (~0.03 mg/mL) and high chemical instability that affects its bioavailability [120,121].

After ingestion, resveratrol undergoes rapid absorption [122]. At a low oral dose (25 mg), the absorption rate is high (~75%), being absorbed by various cell types, but it is not known exactly whether this rate of absorption is maintained at higher doses [123]. However, in dose-escalation studies, resveratrol showed linear pharmacokinetics, even at high doses [124]. After oral administration, resveratrol is absorbed in the intestine by passive diffusion, and once in the bloodstream, is absorbed in the liver by passive diffusion or receptor-mediated transport [120,121,125]. Then, resveratrol can undergo phase II metabolism in the liver, leading to glucuronide–resveratrol and sulfate–resveratrol derivatives, or it can be found in the blood as a free molecule, 90% in the form of complexes, being attached in a non-covalent manner to proteins, such as albumin and lipoproteins (especially LDL), and a small proportion existing in the form of a free fraction of uncomplexed resveratrol [122,126,127]. At the cell membrane level, the complexes will dissociate, following the interaction of albumin and LDL with specific receptors, leaving resveratrol free to enter the cells [121,123,127]. Of the more than 20 metabolites of resveratrol identified in humans and animals, the glucuronide and sulfate conjugates from phase II metabolism are the most abundant, with plasma levels higher than ingested resveratrol [120].

In addition to its rapid metabolism, resveratrol also undergoes rapid excretion, with 75% of the total resveratrol consumed being excreted. Two human studies showed that after the oral administration of 25 mg of resveratrol, the maximum concentration of resveratrol in circulating plasma was below 10 ng/mL, 0.5 h after the oral dose [122,127]. These results show that despite the rapid absorption of resveratrol, its plasma levels are low due to its rapid metabolism [122]. To conclude, the very low oral bioavailability of resveratrol (less than 1–2% of the dose in humans and around 40% in rats) is associated with several factors, such as poor water solubility, which affects its absorption; the high permeability of the intestinal membrane; isomerization due to light exposure; auto-oxidation; and rapid and extensive pre-systemic metabolism [125,127,128,129,130].

Although numerous in vitro studies have shown a wide variety of biological activities associated with resveratrol, these effects cannot be extrapolated in vivo. Therefore, animal studies and clinical trials have not shown similar efficacy for this molecule, as the tissue distribution of resveratrol is very low [122,126,127]. However, resveratrol has also been shown to be efficient in vivo, despite its low bioavailability. The efficacy of resveratrol in vivo may be due to the conversion of its conjugated forms to resveratrol in the liver; to the enterohepatic recirculation of its metabolites, followed by deconjugation and reabsorption; or to the activity of its metabolites [127]. Although it is not known exactly whether the efficacy of resveratrol is due to the compound itself or its metabolites, resveratrol has been shown to be more active than its metabolite, resveratrol monosulfate, in two human bladder cancer (HBC) cell lines, showing greater anti-tumor effects than resveratrol monosulfate and producing a better safety profile in vitro [124]. In contrast, evaluating the effect of resveratrol and its metabolites on the gut barrier and microbiota in a CD-1 mouse model, it was observed that its metabolite, resveratrol-3-O-sulfate, better regulates gut microbial growth and provides superior gut barrier function than resveratrol [124]. Another intensively studied resveratrol metabolite, dihydro-resveratrol, has been shown to be a more effective antioxidant than the vitamin E analog, Trolox [120,131]. In terms of piceatannol, studies show that it has similar biological effects to resveratrol, or is even stronger than its precursor [132]. It has been found in large amounts, as a resveratrol metabolite, in plasma, skin, and liver tissue after the administration of resveratrol in mouse models [120].

Resveratrol bioavailability is increased by gastric juices, so it is recommended to take it with meals [133]. In addition, the circadian rhythm and the type of meal may influence bioavailability [123,134]. Therefore, in order to increase the bioavailability of resveratrol, the best time to administer it turned out to be in the morning [134]. There are wide margins of safety and non-toxicity. Lower doses have a beneficial effect, while higher doses (2 g/day or more) can be associated with a number of side effects, such as diarrhea, nausea, abdominal pain, hypersensitivity, or frontal headache [121,123]. The best dose range, for an actual clinical benefit in vivo, is between 250 mg and 500 mg/day [135].

One of the strategies that may improve the pharmacokinetics and bioavailability of resveratrol is the synergism with other phytochemicals, such as piperine. Thus, the co-administration of resveratrol and piperine has improved the bioavailability of resveratrol by inhibiting its rapid metabolism [126,130]. The use of polydatin, a compound that is extracted from the roots of the *Polygonum cuspidatum* Sieb. et Zucc. plant and differs from resveratrol by the presence of one molecule of glucose—which makes the compound more water-soluble and, consequently, more bioavailable than resveratrol—has also been discussed [121]. It has also been considered to increase the bioavailability of orally administrated resveratrol by using alternative routes of administration, such as inhalers and transdermal, buccal, and nasal–brain routes, obtaining promising results [124].

Other measures that may improve the pharmacokinetics of resveratrol, and therefore bioavailability, have focused on innovative delivery systems, such as nanoemulsions, nanosuspensions, dendrimers, liposomes and nanoliposomes, solid lipid nanoparticles, and polymeric nanoparticles [125,126,130].

### 3.3. Sulforaphane, Indole-3-Carbinol, and 3,3′-Diindolylmethane

The consumption of cruciferous vegetables, such as broccoli (*Brassica oleracea* var. *italica* Plenck), cabbage (*Brassica oleracea* var. *capitata* L.), brussels sprouts (*Brassica oleracea* var. *gemmifera* Zenker), cauliflower (*Brassica oleracea* var. *botrytis* L.), and kale (*Brassica oleracea* var. *viridis* DC. L.) has been associated with anticancer and antioxidant effects. Considerable evidence shows that glucosinolates (GLSs) are the main phytochemicals in cruciferous vegetables that contribute to their health effects [136]. GLSs are relatively inactive and necessitate hydrolysis by plant endogenous myrosinase (MYR) to deliver a variety of bioactive compounds, such as isothiocyanates (ITCs) and indoles. Neutral pH conditions are favorable for the formation of ITCs [137,138]. GLSs and the enzyme MYR are stored in different compartments of plant cells, requiring plant tissue to be damaged for cellular breakdown to occur and MYR to be released and act on GLSs. Therefore, the processing of cruciferous vegetables (i.e., by mastication, cutting, chopping) has an important impact on the bioavailability of GLSs and their hydrolysis products [139,140]. Furthermore, MYR tends to be denatured when cooking cruciferous vegetables, particularly in conditions of increased temperature (>80 °C) and prolonged cooking [136]. In this context, recent research has indicated steaming to be a cooking method more appropriate than boiling in increasing the bioavailability of ITCs from cruciferous vegetables [141,142]. Interesting results are also provided by a study showing that when MYR in cruciferous vegetables is denatured by heating, the supplementation of exogenous MYR can improve the conversion of GLSs to ITCs. More precisely, the study reported that the addition to cooked broccoli of an active source of MYR, in the form of powdered mustard seeds, increased over four times the bioavailability of the ITC sulforaphane (SFN) compared to the bioavailability of SFN in cooked broccoli consumed alone [143].

Nevertheless, even if MYR is inactivated by the thermal treatment of cruciferous vegetables, the ingested GLSs are able to reach the colon, where they can be metabolized by MYR-producing gut bacteria, generating hydrolysis products such as ITCs, which are absorbed or/and excreted [137,144,145]. The hydrolysis of GLSs by the human microbiota has been reported to be highly variable and diverse, a phenomenon that may be attributed to differences in microbiota composition between individuals [146].

Furthermore, the consumption of cruciferous vegetables in their raw form seems to be of interest in order to ensure a better intake of GLS hydrolysis products. Conaway et al. reported that the bioavailability of ITCs from fresh broccoli was about three times higher than that from cooked broccoli, in which MYR is inactivated [147]. Indeed, if MYR remains active in the ingested cruciferous vegetable, it will hydrolyze most GLSs in the small intestine and generate breakdown products that are absorbed at this level [137].

Concerning the assimilation by the body of the GLS hydrolysis products, absorbed ITCs are conjugated to glutathione, with the involvement of glutathione-S-transferase (GST) enzymes, and metabolized via the mercapturic acid pathway [138]. The polymorphisms of genes coding for GST may have an important effect on ITC metabolism, leading to interindividual variations in the benefits from exposure to these compounds. For instance, individuals carrying deletions in both GST M1 and GST T1 genes may show a more rapid elimination of ITCs, requiring a high intake of cruciferous vegetables in order to capitalize on their positive health effects [148]. As for the metabolism of indoles, molecules such as indole-3-carbinol (I3C) principally undergo oxidative metabolization to indole-3-carboxaldehyde and indole-3-carboxylic acid. The quantification of ITC and indole metabolites in human urine and plasma may serve as an approach to characterize the intake of bioactive compounds from cruciferous vegetables [149,150]. Indeed, it has been demonstrated that the urinary elimination of mercapturic acids after the consumption of cooked cruciferous vegetables accounts for a maximum of 20% of the ingested GLSs. If the vegetables are consumed in the raw form, the rate can reach 88% [137].

To date, the most extensively studied ITCs and indoles are SFN and I3C, respectively. SFN is the precursor of glucoraphanin, the main GLS in broccoli, accounting for about 80% of the total yield [73]. Glucobrassicin is also an important GLS in broccoli [150]. The cleavage of glucobrassicin by MYR generates predominantly I3C. In the acidic conditions at the gastric level, I3C further forms a mixture of dimers, linear and cyclic trimers, and higher oligomers, with 3,3′-diindolylmethane (DIM) being the major condensation product [149,151]. Between 20 and 40% of the ingested I3C is converted to DIM [40]. In fact, several studies have suggested that the health effects of I3C can be mainly attributed to DIM [151,152]. I3C, as well as its acid condensation products, are absorbed at the intestinal level and then distributed into several well-perfused tissues, where they exhibit their biological activities [153].

Currently, SFN, I3C, and DIM are considered promising cancer chemopreventive compounds. I3C is also recognized to have biological properties such as the inhibition of inflammation and angiogenesis, decreases in proliferation, and the promotion of tumor cell death [154].

#### 3.3.1. Chemopreventive Activity and Epigenetic Role

There is much evidence to connect the chemopreventive properties of I3C, DIM, and SFN with epigenetic mechanisms [155]. Several studies suggest that, at least in part, the chemopreventive effects of I3C are due to the downregulation of class I HDAC isoenzymes (HDAC1, HDAC2, HDAC3, and HDAC8) by DIM. Decreased HDAC expression leads to the increased expression of the pro-apoptotic Bcl-2 (B-cell lymphoma 2)-associated X (Bax) protein, CDKNs p21, and p27 followed by the arrest of the cell cycle and increased rate of apoptosis. For this reason, HDAC inhibition may be a novel epigenetic mechanism for cancer prevention by DIM [41].

SFN may target the aberrant hypermethylation status by downregulating the expression of DNMT1 and DNMT3a in breast cancer cells [42].

Cyclin D2 is a major regulator of the cell cycle and its hypermethylation is correlated with prostate cancer progression. SFN is capable of decreasing the expression of DNMT1 and DNMT3b and epigenetically modulating cyclin D2 expression, acting as a prostate cancer chemopreventive agent [43].

I3C and DIM modulate the expression of several miRNAs and lncRNAs [82,83]. Thus, DIM increases the expression of tumor suppressor microRNAs, such as let-7a-e, miRNA-15a, miRNA-16, miR-27b, miR-30e, miR-31, miR-34a, miR-124, miR 200 a, miR 200b, miR 200c, miR-219-5p, and miR-320, and decreases the expression of oncogenic miR19a, miR19b, miR92a-2, miR 106a, miR 181a, miR 181b, miR 210-3p, miR 221, and miR 495 [40,44,45].

#### 3.3.2. Effect of Estrogen Analog and Anticarcinogenic in Mammary Tumor Cells

I3C is capable of arresting the growth of human tumor cells in the G1 phase of the reproductive cell cycle [156]. I3C is also a potent inducer of cytochrome P450 enzymes, including CYP1A1, CYP1A2, and CYP1B1 [157,158]. These phase I metabolizing enzymes are involved in the oxidative metabolism of estrogens. I3C and DIM can alter endogenous estrogen metabolism by increasing the 2-hydroxylation reaction, resulting in an increase in the 2-OH:16-OH ratio relative to the estrogen metabolites [159]. The metabolites of these hormones can inhibit or stimulate the onset of hormone-sensitive neoplasms [160]. Several studies have demonstrated that estrone 2 (2OHE1) tends to inhibit the growth of the neoplasm, whereas estrone 16 (16OHE1) promotes tumor growth [161]. The individuals with estrone 2 prevalence are more protected than those with higher levels of estrone 16. Clinical studies have shown that the estrone 2/estrone 16 ratio is an important marker regarding the risk of breast cancer. When this ratio is lower than unity, there are severe clinical forms, while when this ratio is higher than three, the consequences are more favorable [162]. Other products resulting from estrone and estradiol conversion are 2-hydroxylated estrogens, such as 2-hydroxyestrone and 2-hydroxyestradiol, which show anticancer properties that equate them to antiestrogens, targeting several aspects of cancer cell cycle survival and regulation, including cyclin-dependent kinase activities, caspase activation, estrogen metabolism, and estrogen receptor signaling [163,164].

The positive effects of I3C and DIM are related to the fact that both are capable of modifying the estradiol hydroxylation receptor site, resulting in the diminution of 16-α-hydroxyestrone production in favor of 2-hydroxyestrone. I3C and DIM are also involved in the stimulation of liver detoxifying enzyme production, capable of neutralizing and degrading the harmful metabolites of estrogens and xenoestrogens, assimilated as environmental or food pollutants [165,166].

#### 3.3.3. Anticancer Activity

The SFN also exhibits anticancer action by controlling the progression of tumorigenesis. In non-small cell lung cancer (NSCLC), the SFN is able to attenuate the signaling pathway of EGFR, suggesting an anticancer mechanism of action [46]. As a whole, it has shown multiple effects, including the arrest of cell growth, differentiation, and apoptosis, as recently demonstrated in the case of prostate neoplasms [47].

SFN inhibits the proliferation, in vivo, of breast cancer cells, while in normal cells the effect is insignificant. Cancer cells are characterized by the high expression of telomerase. Treatment with SFN inhibits the catalytic subunit of human telomerase reverse transcriptase (hTERT) [167]. At the same time, scientific studies have shown interference in DNA methyltransferase (DNMT) activity, in particular DNMT1 and DNMT3a, which have been reduced in breast cancer cells treated with SFN, suggesting that this compound may be able to repress hTERT through specific epigenetic pathways. Furthermore, the downregulation of hTERT expression facilitates the induction of cell apoptosis in breast cancer cells, paving the way for approaches aimed at the SFN-mediated prevention of this neoplasia and as preventive nutraceuticals [48].

#### 3.3.4. Anti-Inflammatory Activity

Inflammation is usually associated with chronic disease and cancer. It is well known that NF-κB is a major transcription factor involved in the regulation of the expression of many pro-inflammatory genes, such as COX-2 and iNOS. I3C and DIM exert anti-inflammatory effects by the downregulation of COX-2, iNOS, CXCL5, and IL-6 expression, which may be mediated by reductions in NF-κB activation [168].

In conclusion, the GLSs present in cruciferous vegetables have beneficial effects on general health and are also potential anticancer agents, due to their antioxidant and detoxifying properties and epigenetic mechanisms, including the modification of CpG (cytosine–phosphate–guanine) methylation, which occurs predominantly in cancer-related genes, the regulation of histone modification, and changes in miRNA expression [169,170].

The daily dosage of SFN demonstrated to provide beneficial health effects is around 20–40 mg [171]. Furthermore, the recommended daily dosage for I3C ranges between 200 mg and 900 mg per day and for DIM between 25 mg and 450 mg per day, respectively. The use over time must include both urinary and blood hormone monitoring, including, in the urine, the observation of the relationship between estrone 2 and estrone 16 and, at the hematic level, of the total estrone and estradiol and total and free testosterone and androstenedione, so as to constantly adapt the therapy. A diet rich in cruciferous vegetables seems to provide SFN, I3C, and DIM in sufficient amounts for the prevention of many types of cancer, including those that are hormone-related, such as breast, ovary, uterus, and prostate neoplasms [172,173,174]. In contrast, to achieve therapeutic concentrations of SFN, I3C, and DIM, the intake of these compounds in the form of dietary supplements seems to be required [40,175].

The exploitation of SFN by the nutraceutical industry has faced some challenges because this ITC shows high lipophilicity, low aqueous solubility, and poor stability due to sensitivity to oxygen, heat, and alkaline conditions. However, the use of nanotechnology has allowed the increase in the aqueous solubility and bioavailability of SFN through the development of formulations such as polymeric nanoparticles, magnetic nanoparticles, micelles, liposomes, and carbon dots [175].

Likewise, the low thermal- and photostability of I3C and DIM represent important challenges for the nutraceutical application of these compounds. One approach to overcome this issue has been proposed by Luo et al. (2013), who showed that the encapsulation of I3C and DIM in zein/carboxymethyl chitosan nanoparticles can protect these bioactives against temperature- and light-induced degradation [176].

### 3.4. Astaxanthin

Astaxanthin (3,3′-dihydroxy-β, β′-carotene-4,4′-dione) (ASX) is a red-orange pigment, a xanthophyll carotenoid, and a member of the macro-family of carotenoids [177]. Synthesized in appropriate quantities by microalgae—*Haematococcus lacustris* (Gir.-Chantr.) Rostaf., *Chromochloris zofingiensis (Donz) Fucikova and L. A. Lewis*, *Chlorococcum sp.*, and *Phaffia rhodozyma* M.W. Mill., Yoney. and Soneda—ASX enters the food chain through crustaceans and predatory fish such as salmon, in whose meat it can easily reach 5–10 mg/kg [178].

ASX has antioxidant potential, as well as anti-inflammatory and antineoplastic activities, acting as an antioxidant and reducing oxidative stress, thereby preventing protein and lipid oxidation and DNA damage. Having antioxidant action, it helps to maintain the functionality of tissues and systems, promoting better overall homeostasis [177].

ASX affects tumor growth in different types of cancers. Several studies have demonstrated that ASX is able to resensitize gemcitabine-resistant human pancreatic cancer cells to gemcitabine [49]. ASX increases DNMT3a expression at low concentrations, but at high concentrations decreases the expression of DNMT1, 3a, and 3b and attenuates NAD(P)H Quinone Dehydrogenase 1 (NQO1) expression via the Nrf2/KEAP1 pathway, reducing cell viability in prostate and skin cancer cells [50,51]. ASX has also the ability to reduce tumor growth in prostate cancer by increasing the expression of tumor suppressor microRNAs, miR-375 and miR-478b [52]. In breast cancer, ASX negatively affects cell viability [179,180], due to apoptotic and autophagic effects that allow it to kill the cancer cells without affecting normal cells [181].

In colorectal cancer (CRC), ASX has demonstrated anti-migratory and anti-invasive activity by increasing miR-29a-3p and miR-200a expression, suppressing MMP2 and ZEB1 expression, resulting in the repression of the epithelial–mesenchymal transition (EMT) of CRC cells [53]. Regarding lung cancer, NSCLC accounts for the majority of lung cancer-related deaths [182]. There are few studies to show the effects of ASX against NSCLC or other lung cancers in vivo. In vitro, ASX is able to reduce the viability of NSCLC cells in a dose-dependent manner [183,184]. Moreover, ASX enhances apoptosis and decreases cell proliferation. ASX is able to enhance the cytotoxicity of the drugs with clinical activity in NSCLS, such as erlotinib, a selective epidermal growth factor receptor (EGFR) tyrosine kinase inhibitor. The co-administration of erlotinib and ASX has increased cytotoxicity and inhibited cell growth in NSCLC cells, associated with the downregulation of xeroderma pigmentosum complementation group C (XPC) expression [184]. The overexpression of thymidylate synthase (TS) usually causes resistance to antitumor treatment, especially pemetrexed used in advanced NSCLC forms. ASX treatment decreases TS expression, both alone and in combination with pemetrexed. Moreover, ASX administration together with mitomycin C significantly reduces Rad51 expression, which exhibits high levels in chemoresistant carcinoma [54,55].

All these in vitro findings suggest that ASX may improve the efficacy of standard treatments in lung cancer. Some studies have also suggested that ASX could be used to treat gastric cancer, based on its role in necroptotic signaling [185].

Despite its biological activities, ASX has very low bioavailability, similar to other carotenoids [55,186]. When astaxanthin is administered orally, the bioavailability varies between 10 and 50% of the given dose [187]. The very poor bioavailability of ASX is due to dissolution limitations in gastrointestinal fluids and also to the saturated capacity of incorporation into bile micelles, which limits its absorption [188,189]. Being a very lipophilic compound, it has extremely low water solubility, which prevents its dispersibility and causes a low absorption rate [55,190]. After ingestion, ASX mixes with bile acid, forming micelles in the small intestine, partially absorbed by intestinal mucosa cells, which will incorporate astaxanthin into chylomicrons [178]. After their release into the lymph within the systemic circulation, chylomicrons with ASX are digested by lipoprotein lipase, ASX is assimilated with lipoproteins and transported to tissues, and chylomicrons remnants are quickly removed by the liver and other tissues [178]. In nature, astaxanthin is predominantly found in the form of mono- and diesters, being, respectively, esterified with one or two units of fatty acids in hydroxyl groups, or in the form of carotenoproteins when conjugated with proteins [187]. Recent research shows that ASX bioavailability varies depending on its molecular structure, origin, and isomerization [187,191,192]. Therefore, in the case of natural astaxanthin in esterified form, bioavailability is improved by facilitating its incorporation into mixed micelles in the lumen, where unsaturated lipids are released by the action of bile salts and pancreatic lipases before free astaxanthin is absorbed by intestinal mucosal cells [187]. Of the esterified forms of ASX, monoesters have shown significantly higher bioavailability than astaxanthin diesters [191]. Regarding the difference in bioavailability arising from isomerization, the spatial arrangement of atoms in the case of the 3S,3′S stereoisomer has been shown to increase the bioavailability of astaxanthin compared to the 3R,3′S and 3R,3′R isomers [193,194]. While synthetic ASX is a racemic mixture of the three stereoisomers (3S,3′S; 3R,3′S; 3R,3′R), naturally occurring ASX is in the form of the isomer 3S,3′S [194]. Thus, for the most efficient 3S,3′S stereoisomer, the main primary source is the green alga *Haematococcus lacustris* (Gir.-Chantr.) Rostaf., but also other sources such as *Paracoccus carotinifaciens* Tsubokura et al. or *Salmo salar* L., while the 3R,3′S and 3R,3′R stereoisomers, respectively, have primary sources such as distilled petroleum and the yeast *Phaffia rhodozyma* M.W. Mill., Yoney. and Soneda, respectively [193,194]. In addition, due to its highly unsaturated molecular structure, ASX has reduced chemical stability during processing, storage, and digestion, being easily degraded in both acidic and alkaline environments and also under UV light, oxygen, or heat action [55,195,196]. The bioavailability of ASX has been reported to be increased in humans by its incorporation into lipid formulations of various compositions, and this may be due to the presence of conjugated bile salt and its ability to form bile salt micelles [189]. The combination of ASX with edible oils has led to both high bioavailability and stability [178,193]. Enhanced ASX absorption was demonstrated in a study with a combination of ASX and fish oil, which succeeded in promoting hypolipidemic/hypocholesterolemic effects in plasma and its increased phagocytic activity of activated neutrophils compared to ASX and fish oil separately [193,197]. Moreover, the administration of *Haematococcus lacustris* (Gir.-Chantr.) Rostaf. biomass dispersed in olive oil has shown increased bioavailability and enhanced antioxidant properties in ASX, both in rat plasma and in liver tissues [178,193]. In addition to the dietary fat content, another factor with a major influence on astaxanthin absorption is smoking. Thus, the bioavailability of astaxanthin in smokers is reduced by 40% [193].

In order to improve the water solubility, stability, and bioavailability of ASX, several delivery systems have been developed, such as complex coacervation, liposomes, emulsions and nanoemulsions, microparticles, and polymeric nanoparticles [196,198,199]. Since lipid-based nanoparticles, such as liposomes, solid-lipid nanoparticles, and niosomes have limitations, such as poor water solubility and permeability, instability, rapid metabolism, and poor oral bioavailability, polymeric nanoparticles have begun to be used to overcome these limitations. Thus, after encapsulation in polymeric nanoparticles made from biodegradable natural polymers, such as polysaccharides and proteins, water solubility, stability, and absorption in the human body were enhanced [190,199]. In terms of nanoemulsion-based delivery systems, they provide improved physical stability and increase water dispersibility and bioavailability [184].

### 3.5. Quercetin

Quercetin is a flavonoid belonging to the flavonols group, present in a large variety of fruits—apples (*Malus domestica* (Suckow) Borkh.), grapes (*Vitis vinifera* L.), olives (*Olea europaea* Hoffmanns. and Link L.), citrus fruits such as oranges (*Citrus sinensis* (L.) Osbeck), and raspberries (*Rubus idaeus* L.)—vegetables—tomatoes (*Solanum lycopersicum* L.), onions (*Allium cepa* L.), broccoli (*Brassica oleracea* var. *italica* Plenck), and capers (*Capparis spinosa* L.)—drinks (tea and red wine), and herbal extracts [128]. In nature, quercetin is not present in the isolated form but is the aglyconic component of some glycosides, including rutin and quercitrin. In this form it abounds, in particular, in extracts of horse chestnut (*Aesculus hippocastanum* L.), *Gingko biloba* L., marigold (*Calendula officinalis* L.), hawthorn (*Crataegus monogyna* Jacq.), chamomile (Matricaria recutita L. and *Chamaemelum nobile* L.), lovage (*Levisticum officinale* W. D. J. Koch), and St. John’s wort (*Hypericum perforatum* L.) [200,201]. Quercetin is a natural anti-inflammatory, antioxidant and anti-cancer compound with many abilities, its most important functions being mentioned in Table 3.

Many in vitro and in vivo studies have demonstrated the anticancer effects of quercetin against breast, prostate, kidney, colorectal, ovarian, gastric, nasopharyngeal, and pancreatic cancer. The antitumor effects include the inhibition of angiogenesis proliferation, the inhibition of the cell cycle, and tumor metastasis prevention [56].

Quercetin is capable of increasing the pro-apoptotic molecules BAX, caspase-3, caspase-9, and p53 and stimulating the mitochondrial apoptosis pathway, resulting in increased proapoptotic effects [57,59]. Another important feature of quercetin is related to the arrest of the cell cycle in the G1 phase by activating p21 and decreasing D1/Cd4 and E/Cdk2 ratios [211,212]. Several studies have demonstrated that quercetin can inhibit carcinogenesis and metastasis in cancer and is capable of stabilizing p53, a key molecule in cancer therapy involved in cell death and survival regulation [60].

In gastrointestinal cancer (GC), the genes encoding for the proteins urokinase plasminogen activator (uPA) and uPA receptor (uPAR) are strongly associated with this type of cancer, being a crucial pathway for tumor invasion. Quercetin has the ability to decrease the expression of these genes, strongly associated with the suppression of cell viability, migration, and invasion. Likewise, quercetin has antimetastatic effects in GC by interfering with uPA/uPAR systems, AMPKα, NF-kβ, ERK1/2, and PKC-δ regulation [61]. In patients with CRC carrying the KRAS mutant gene, quercetin decreases cell viability and increases apoptosis by AKT pathway repression and the activation of the c-Jun N-terminal kinase (JNK) pathway in mutant KRAS cells [62].

In prostate cancer, quercetin inhibits the expression of androgen receptor (AR) and AR-mediated PSA expression at the transcriptional level with the inhibition of tumor progression. Quercetin can suppress survival protein Akt and enhance prostate cancer apoptosis in a dose-dependent manner [63].

Quercetin decreases IGF1 levels and increases IGFBP3, which is associated with an increase in proapoptotic effects and a decrease in anti-apoptotic proteins BCL2 and BCL-XL [64].

Src is a non-receptor tyrosine kinase that is deregulated in many types of cancer. Quercetin has an anti-NSCLC effect in lung cancer by inhibiting the Src-mediated Fn14/NF-κB pathway [213].

The epigenetic mechanisms associated with quercetin are the suppression of Janus kinase 2 (JAK2) with the inhibition of the proliferation, invasion, and migration of cancer cells [65]. Quercetin can also enhance apoptosis through its DNA-demethylating activity. Quercetin has an inhibiting effect on class I HDAC expression in leukemia cells due to increased proteasomal degradation [66]. Quercetin turns out to be a valid nutraceutical that can help reduce the formation of free radicals and pro-inflammatory substances, proving to be a valuable aid for human health.

Quercetin has also been shown to modulate the expression of microRNAs in different types of cancer by increasing the expression of tumor-suppressive miR-let-7, miR-15a, miR-16, miR-16, miR-22, miR-26, miR-200b-3p, miR-142-3p, miR-146a, miR-217, and miR-330 and decreasing the expression of oncogenic miR-27a, miR-155, miR-21, miR-19b, miR-148c [58].

The bioavailability of quercetin is generally poor and characterized by high interindividual variability, which could explain the conflicting results on quercetin bioactivities reported in various studies [214,215]. Pharmacokinetic studies indicate a low absorption of quercetin, with less than 1% of quercetin being absorbed in humans following oral administration [214,216]. The absorption of quercetin is related to its solubility in the vehicle used for administration [214,217]. Thus, the low solubility of quercetin in water, gastric fluids, and small intestine fluids will limit its absorption in the body [216,218]. The absorption of quercetin depends on its chemical structure. Thus, while quercetin aglycone is absorbed in both the stomach and small intestine, glycosylated forms of quercetin are not absorbed in the stomach and will be absorbed only in the small intestine, after deglycosylation, as quercetin aglycone. Quercetin biotransformation occurs by small intestinal and hepatic xenobiotic metabolism, which consists of three phases: phase I modification, phase II conjugation, and phase III elimination. Quercetin is rapidly eliminated via feces and urine. In addition to poor absorption, another factor limiting the bioavailability of quercetin is its hepatic biliary excretion, a significant proportion of absorbed quercetin being directed to biliary elimination and not to circulation [214,215].

Other factors that may affect quercetin bioavailability include the food matrix, nondigestible fiber, dietary fat, the presence of sugar moieties, and the botanical origin of quercetin. The results of a randomized crossover study, in which six women ingested the same amount of quercetin either in cereal bars or hard capsules, showed that the bioavailability of quercetin is higher when the quercetin aglycone is consumed as a whole food component [218,219]. In a study of rats, the influence of nondigestible oligosaccharides on the bioavailability of quercetin was examined. The co-administration of quercetin with short-chain fructooligosaccharides has been shown to improve the bioavailability of quercetin, as microbial degradation of the quercetin aglycone in the large intestine has been inhibited, thereby promoting the absorption of the quercetin glycoside [214,218]. Since quercetin aglycone is lipophilic, its co-ingestion along with fat has been able to increase the absorption of quercetin by incorporating it into micelles. This was observed in a study using pigs, but the improvement in the quercetin bioavailability in the case of fatty food ingestion was also demonstrated in another in vivo study in humans [214,218,219]. The bioavailability of quercetin may also be influenced by the presence or absence of the glucoside moiety, with studies in pigs showing the increased bioavailability of quercetin glycoside compared to quercetin aglycone, most likely due to the preferential absorption of quercetin glucoside, which is more water-soluble than quercetin aglycone [214]. In addition, the bioavailability of quercetin glycosides may be influenced by the type of sugar moiety [219]. Another factor on which quercetin bioavailability depends is its botanical origin. Thus, comparing the bioavailability of different forms of quercetin derivatives from onions, apples, and tea, it was observed that quercetin glycosides from onions had the highest bioavailability. The bioavailability of quercetin in humans may also be affected by health status, gut microbiota, genetic factors, and oxidative stress [218]. Various approaches have been used to improve the water solubility and bioavailability of quercetin, such as encapsulation in nanoparticles, emulsions and nanoemulsions, hydrogels, cyclodextrin complexation, size reduction (nanosuspension, nanocrystals, nanorods), co-crystallization, and amorphous solid dispersions [216,218,220].

### 3.6. Epigallocatechin-3-Gallate

EGCG is the most abundant catechin in tea, especially in green tea (*Camellia sinensis* L.). EGCG is a polyphenol with antioxidant and anti-inflammatory action. Besides tea, it is also found in smaller quantities in other foods, such as carob (*Ceratonia siliqua* L.) flour, apples (*Malus domestica* (Suckow) Borkh.), blackberries (*Rubus plicatus* L. Weihe and Nees), raspberries (*Rubus idaeus* L.), pistachios (*Pistacia vera* L.), prunes (*Prunus domestica* L.), peaches (*Prunus persica* (L.) Batsch), and avocados (*Persea americana* Mill.). From the tea plant, for production, the leaf bud and the two adjacent leaves are used together with their stem. Green tea is very rich in polyphenols, and among them, EGCG is the most-studied active ingredient with the highest antioxidant activity [221].

In the case of polyphenols, the antioxidant action is achieved through the oxidation of polyphenols to quinones (which, although toxic to the body, polymerize after their formation, so they are no longer absorbed) and the reduction in the relative substrates. The green tea catechins are capable of modulating epigenetic processes, reversing DNA methylation in the tumor suppressor genes, and increasing their relative transcription. They also modulate DNA methylation, mitigating the effect of DNMT1 (direct enzymatic inhibition, indirect enzymatic inhibition, reduced DNMT1 expression, and reduced translation) [67]. Another epigenetic mechanism would then be related to the redox properties of green tea catechins and their ability to inhibit histone deacetylase (HDAC) [68].

In vivo studies have also shown that the high consumption of green tea, and therefore a high intake of EGCG, has the ability, compared to placebo groups, to decrease the methylation of CDX2 and BMP-2 in gastric carcinoma, with effective epigenetic modulation [69].

EGCG, and tea polyphenols in general, are capable of mediating the epigenetic induction of metalloproteinase inhibitors (TIMP), such as TIMP-3, whose levels have a key role in suppressing the gelatinolytic activity of MMP-2 and MMP-9, involved in the metastatic process. Therefore, EGCG is considered a modulator of metalloproteinase activity, with benefits at oncological levels [70].

EGCG also has the ability to inhibit acute promyelocytic leukemia (APL) by inhibiting cell proliferation and promoting apoptosis [222]. In cell culture and animal models of prostate, breast, skin, liver, bladder, lung, and digestive tract cancer, EGCG induces the inhibition of cell proliferation and apoptosis by affecting the MAPK/ERK pathways and growth factors IGF1, IGF, and IGFBP-3. By inhibiting PI3K/AKT/p-BAD, a cell survival pathway, EGCG controls apoptosis. Moreover, EGCG is able to inhibit angiogenesis, invasion, and VEGF [69]. EGCG is an important regulator of cancer-associated microRNAs and upregulates miR-16, miR-210, and miR-330 and decreases miR-21 and miR-98-5p expression in liver, prostate, and lung cancer [71].

Despite the numerous health-promoting properties of EGCG demonstrated by in vitro and in vivo studies, its use by humans poses challenges due to poor systemic bioavailability [223]. After ingestion, EGCG requires effective intestinal absorption to further exhibit its biological activities. However, EGCG seems to be poorly absorbed by the body, reaching only a reduced concentration in the plasma and then rapidly (<8 h) becoming undetectable in the systemic circulation [224]. Overall, it has been estimated that only about 1% of the orally-consumed EGCG is absorbed into the circulatory system in order to further reach target organs [225]. For instance, research on human subjects has reported a plasma concentration of EGCG as low as 0.15 μM following the consumption of two cups of green tea [226]. Such a result may be due, at least in part, to EGCG being hydrolyzed by esterases in the saliva but also due to this compound being degraded under the alkaline pH conditions of the duodenum. In fact, the EGCG molecule undergoes autoxidation at alkaline pH, with the formation of oxidative products [227]. In addition, EGCG is extensively decomposed by intestinal microorganisms [228]. Indeed, only a minor proportion of the ingested EGCG is absorbed in the upper gastrointestinal tract. The remaining fraction of EGCG transits from the small to the large intestine, where it undergoes metabolism by local microbiota, leading to the formation of various catechin ring-fission products [229]. These latter metabolites can be excreted into urine or reabsorbed into the systemic circulation and further act as bioactives [230,231]. Anti-oxidative, anti-inflammatory, and anti-cancer effects have been reported for catechin ring-fission metabolites, suggesting that these compounds may actually contribute to some of the health benefits attributed to EGCG [224].

As concerns the intestinal absorption of EGCG, this process seems to show low efficiency, as EGCG lacks specific receptors for its absorption and is carried by passive diffusion (e.g., paracellular diffusion, transcellular diffusion) across epithelial cells. Following absorption, EGCG undergoes a phenomenon of active outflow, mediated by components of the efflux transport system (e.g., P-glycoprotein, multidrug resistance-associated proteins, breast cancer resistance proteins) that actively efflux intracellular EGCG to the extracellular intestinal space [225,232]. At the level of the small intestine and liver, EGCG is metabolized by phase II enzymes, releasing glucuronidated, sulfated, and methylated conjugates. EGCG metabolites are excreted through both bile and urine. EGCG can be further reabsorbed from the intestine through the enterohepatic recirculation process [233,234,235].

To capitalize on the therapeutic potential of EGCG in humans, despite its reduced bioavailability, high intakes have been suggested (e.g., the consumption of 8 to 16 cups/day of green tea) [236]. Nevertheless, using high doses of catechins may be of concern in the context of their dose-dependent toxic effects. A recent report by the European Food Safety Agency indicated a risk of liver damage following the intake of EGCG in the form of dietary supplements, at doses of 800 mg/day or above [237,238]. In order to manage these issues and improve the bioavailability of EGCG, several approaches have been identified. One approach involves the co-administration of EGCG with other bioactives. For instance, a formulation with ascorbic acid and sucrose has been demonstrated to enhance EGCG bioavailability by increasing its bioaccessibility and intestinal uptake from green tea [239]. Likewise, it has been suggested that the ingestion of EGCG on an empty stomach may improve its systemic absorption [240]. Moreover, the structural modification of EGCG by methylation, acyclization, or glycoside modification seems to allow the management of its premature degradation and reduced absorption rate [228,233]. Finally, one promising approach to protect EGCG against unfavorable gastrointestinal conditions and improve its bioavailability includes the design of nanocarriers. Examples of carriers developed for the nanodelivery of green tea catechins comprise surfactant-based nanovesicles (liposomes, phytosomes, niosomes, bilosomes), polysaccharide nanostructures, protein nanoparticles, nanoemulsions, and nanostructured lipid carriers [225,241].

### 3.7. Lycopene

Lycopene is a non-provitamin A carotenoid, present particularly in tomatoes (*Solanum lycopersicum* L.), but also in apricots (*Prunus armeniaca* S. X. Sun L.), guava (*Psidium guajava* L.), papaya (*Carica papaya* L.), watermelon (*Citrullus lanatus* subsp. *vulgaris* (Schrad.) Fursa), and pink grapefruit (*Citrus × paradisi* Macfad.). The level of ripeness in these fruits influences their lycopene content. For example, the content of lycopene is 50 mg/kg in ripe tomatoes, but only 5 mg/kg in unripe yellow tomatoes [242]. In addition to having a powerful antioxidant action, lycopene can improve the fluidity of the circulating blood mass and reduce the inflammatory response [243].

Carotenoids, as well as their metabolites and oxidation products, improve communication at the level of the intercellular junction gate GJC (Gap Junction Communication), which is considered one of the mechanisms of cancer prevention. GJC is deficient in many forms of cancer and the restoration of this function leads to cell proliferation reduction [244]. Several studies have demonstrated that lycopene is capable of modulating the expression of genes involved in inflammation, apoptosis, and cancer progression and, in this manner, reducing prostate cancer risk [245,246].

The link between lycopene intake and prostate cancer risk has been studied for decades, and the results suggest, in part, that sufficient lycopene intake could reduce the risk of prostate cancer [74]. As concerns the underlying mechanism, an epigenetic one, lycopene downregulates serine/threonine kinase 2 (AKT2) and upregulates miR-let-7f-1, with the inhibition of prostate cancer progression [75]. Usually, prostate cancer involves the silencing of GSTP1 and previous studies have shown that lycopene treatment activated the GSTP1 promoter and downregulated DNMT3a in a PC-3 cell line [73]. A limited number of studies have suggested that lycopene decreases cyclin D1, D3, CDK2, and CDK4, with cell-cycle arrest in G0/G1, and increases the expression of the p53 tumor suppressor [72].

These findings suggest that lycopene could act as a promising anticancer agent and may lower the risk of some types of cancer.

The dosage range varies mainly between 20 and 50 mg/day, but up to 100 mg daily can be reached without safety issues. In fresh and ripe tomatoes, about 90% of lycopene is found in the all-*trans* geometric configuration. Factors such as high temperature, light, and the presence of oxygen lead to the isomerization of lycopene. Therefore, in processed tomato products (e.g., ketchup, tomato concentrate, tomato juice, tomato powder), the forms of *cis*-lycopene dominate [247].

Although the majority of the lycopene in unprocessed food is found in the all-*trans* isoform, human serum and tissues have been reported to contain mainly the *cis* isomers of this carotenoid. A potential mechanism to explain this phenomenon could be an intestinal absorption that is preferential for the forms of *cis*-lycopene [248]. Indeed, evidence from early in vitro and animal studies has suggested that the *cis* isomers of lycopene have greater bioavailability than the all-*trans* form, possibly due to *cis* isomers showing a shorter length, higher solubility in mixed micelles, and/or a lower tendency to aggregate into crystalline structures [249]. However, more recent studies in human subjects have reported that there are no significant differences between *cis*-lycopene and all-*trans* lycopene absorption, indicating heat-induced isomerization or enzymatic isomerization within body tissues as processes that could explain the enhanced *cis* isomeric profile in human serum and tissues [250].

As concerns the intestinal absorption of lycopene, this process occurs either by passive diffusion or via the scavenger receptor class B type 1 protein (SR-B1) transporter. Nevertheless, the intestinal absorption of lycopene seems to have limited efficiency [248]. In the study conducted by Moran et al. (2015), human subjects absorbed only about 24% of the ingested lycopene. Lycopene is mainly stored in the liver, but it can also accumulate within extrahepatic tissues (e.g., adipose tissue, adrenals, skin, kidneys, lungs, prostate, testes, ovaries, and breastmilk) [248,250]. During its initial metabolism, lycopene produces lycopenoids (e.g., APO-10′-lycopenoic acid). Some studies suggest that the health effects of lycopene are actually related to the biological activities of lycopenoids, but more research is required to clarify this aspect [251]. Lycopene is mainly excreted through the feces and in lower amounts through the urine [248].

In recent years, there has been a growing interest in identifying factors that could improve the bioavailability of lycopene from dietary sources such as tomatoes. The mechanical treatment of tomatoes (e.g., by mastication, grinding) appears to be important for lycopene bioaccessibility and hence for its plasmatic bioavailability [252]. Likewise, the heat processing of tomatoes may increase the bioavailability of lycopene in these foods by favoring its *trans*-to-*cis* isomerization. It has also been suggested by several studies that the addition of dietary fats (e.g., olive oil, avocado) to tomato dishes may increase the absorption and consequently the plasma levels of lycopene [253,254]. A recommendation has been issued to add a minimum of 10 g of fat in culinary preparations containing processed tomato products and 15 g of fat in fresh tomato recipes, respectively [248]. The encapsulation in nanoparticles of the lycopene extracted from food sources or biosynthesized also seems to help in solving its bioavailability issues and capitalizing on its nutraceutical potential [255].

Furthermore, there are several factors related to the characteristics of human subjects ingesting lycopene that have been reported to influence the bioavailability of this bioactive compound. Gender, adiposity, body mass index, and smoking habits appear to contribute to about 25% of the variation in serum lycopene concentrations [256]. In addition, there are studies to suggest that certain genetic variants linked to carotenoid metabolism may have an impact on lycopene bioavailability [256,257,258]. For example, the single nucleotide polymorphism rs6564851 in the β-carotene 15,15′-oxygenase-1 (BCO1) gene, which encodes for the BCO1 enzyme responsible for carotenoid cleavage in mammals, has been reported as being significantly associated with changes in lycopene circulating levels [256].

## 4. Conclusions

Epigenetic modifications have a significant role in cancer pathogenesis and incidence. This review provides recent evidence on the anti-tumor effect of nutraceuticals. The selected plant-based nutraceuticals have demonstrated a potential benefit in the reversion of cancer hallmarks due to their ability to modulate gene expression through epigenetic mechanisms. These biocompounds are effective in several biological functions, such as cell cycle arrest, cell proliferation, induction, and the inhibition of apoptosis in tumor cells. It is clear that epigenetic mechanisms are novel targets for the use of nutraceuticals in the prevention and treatment of cancer. The combination of nutraceuticals with chemotherapeutic drugs can enhance the effects of the latter. Nutraceuticals represent a cutting-edge and rapidly evolving segment in the field of health products and the increasingly strong scientific evidence for these molecules supports their widespread use as adjuvants in predictive and preventive medicine. However, more studies are required to fully understand the complexity of the epigenetic mechanisms involved in cancer as well as the role of nutraceuticals, especially in aggressive and invasive cancers showing resistance to conventional therapies. Future research should be focused on personalized epigenetic diets for cancer prevention or treatment approaches in cancer.

## Figures and Tables

**Table 1:** Summary of the gene expression variability and antitumor activity.

Natural Source	Epigenetic Modulation	Gene Targets	Biological Effects	Micro RNAs Regulated	Cancer Types	References
Curcumin						
Turmeric	DNMT1 DNMT3b DNMT3a HDAC1 HDAC4 HDAC7	P65, Sp1, CDK, Her2, NrF2, STAT3, BAX, p38, p53 VEGF IL6 IL23 IL1-β	Chemoprevention, cell growth inhibition, cell-cycle arrest Apoptosis, angiogenesis inhibition	miR-15a↑ miR-16↑ miR-22↑ miR-26a↑ miR-34a↑ miR-145↑ miR-146a↑ miR-200b↑ miR-200c↑ miR-203 ↑ let7↑ miR-19a,b↓ miR-21↓ miR-27a↓ miR-130a↓ miR-186↓	AML Breast Prostate Colon Lung	[19,20,21,22,23,24,25,26,27,28,29,30,31]
Resveratrol						
Black grapes, red wine, plum, peanuts, berries, cocoa powder, dark chocolate	DNMT HDAC	p53 p300 p16 CDK AP1 EGR1 STAT1 STAT3 SIRT1 MAPK Bcl2 hTERT MTA1	Cell growth inhibition, cell-cycle arrest Apoptosis Chemopreventive	miR34a↑ miR 663↑ miR 141↑ miR 200↑ miR17↓ miR25↓ miR92a-2↓	Colon Breast Prostate Lung	[32,33,34,35,36,37,38,39]
Sulforaphane						
Broccoli Cauliflower Cabbage Brussels sprout	DNMT1 DNMT3a DNMT3b HDCA1, 2,3,8	p21 p27 CDKN hTERT EGFR Cyclin D2 Nrf2	Chemopreventive Cell-cycle arrest Apoptosis Cell growth inhibition	miR-let-7a-e↑ miR-15a↑ miR-16↑ miR-27b↑ miR-30e↑ miR-31↑ miR-34a↑ miR-124↑ miR-200a-b-c↑ miR-219-5p↑ miR-320↑ miR-19a↓ miR-19b↓ miR-92a-2↓ miR-106a↓ miR-181a↓ miR-181b↓ miR-210-3p↓ miR-221↓ miR-495↓	Prostate Breast Lung	[40,41,42,43,44,45,46,47,48]
Astaxanthin						
Algae, yeast, salmon, trout, krill, shrimp, and crayfish	DNMT1 DNMT3a DNMT3b	MMP2 ZEB1 EMT EGFR XPC Rad51 NQO1 NRF2/ KEAP1	Chemopreventive Apoptosis Cell growth inhibition Cell proliferation inhibition	miR-29a-3p↑ miR-200a↑ miR-375↑ miR-478b↑ miR-221↓	Pancreatic Lung Prostate Skin	[49,50,51,52,53,54,55]
Quercetin						
Onion, apple, citrus fruits, raspberries Grapes Olives Tomatoes	DNMT3a DNMT3b HDAC1 DNMT1	p53 CD1 p21 PLAU ERK1/2 KRAS BRCA1 BRCA2 IGF1 IGFBP3 JNK AR Bcl2 JAK	Cell growth inhibition Cell proliferation inhibition Chemopreventive Apoptosis Cell-cycle arrest	miR-let-7↑ miR-146a↑ miR-15a↑ miR-16↑ miR-26↑ miR-142-3p↑ miR-200b-3p↑ miR-217↑ miR-330↑ miR-27a miR-21 miR-19b miR-155 miR-148c	Breast Prostate Colon Ovarian Gastric Pancreatic Lung Leukemia	[56,57,58,59,60,61,62,63,64,65,66]
EGCG						
Green tea, carob flour, apples, pistachios, prunes, peaches, avocados	DNMT1 DNMT3a DNMT3b HDCA1	GSTP1 CDX2 BMP2 TIMP3 MMP2 MMP9 IGF, IGF1, IGFBP-3 VEGF p53 Bcl2	Cell growth inhibition Cell proliferation inhibition Chemopreventive Apoptosis Cell-cycle arrest Angiogenesis decreases	miR-16↑ miR-210↑ miR-330↑ miR-21↓ miR-98-5p↓	Liver Breast Prostate Lung Bladder Gastric Colon	[67,68,69,70,71]
Lycopene						
Tomatoes Apricots Guava Papaya Watermelon Pink grapefruit	DNMT3a	GSTP1 AKT2 CDK2 CDK4 p53 CCND1 CCND3	Cell growth inhibition Chemopreventive Cell-cycle arrest Apoptosis	miR-let-7f-1 ↑	Prostate cancer Breast cancer	[72,73,74,75]

↑ increases expression; ↓ decreases expression.

**Table 2:** List of plant-derived bioactives currently in clinical trials on various types of cancer [76].

Plant-Derived Bioactive Compound	Type of Cancer	Primary Outcome Measures	Clinical Trial Identifier
Curcumin	Breast cancer	Tumor proliferation rate	NCT03980509
Sulforaphane	Lung cancer	Prevention of lung cancer in former smokers/bronchial dysplasia index	NCT03232138
Quercetin	Squamous cell carcinoma	Prevention of squamous cell carcinoma in patients with Fanconi anemia/reduction in buccal micronuclei	NCT03476330
Epigallocatechin-3-gallate	Colorectal cancer	Change in methylation from baseline when compared to the control arm	NCT02891538
Lycopene	Metastatic colorectal cancer and skin toxicity	Skin toxicity reduction in metastatic colorectal cancer submitted to therapy with panitumumab	NCT03167268
Mixture of carotenoids, indole-3-carbinol, curcumin, EGCG, caffeine, resveratrol, lycopene, genistein, phytoestrogens	Breast and ovarian cancer syndrome	DNA damage change	NCT05306002

**Table 3:** The most important functions of quercetin.

Quercetin’s Functions	References
Ability to restore tocopherol after its transformation into tocopheryl radical.	[202]
Ability to protect the endogenous antioxidant enzymatic systems, catalase (CAT), superoxide dismutase (SOD2), glutathione peroxidase (GPX), and glutathione reductase (GR).	[203]
Ability to eliminate superoxide anion and limit nitric oxide biosynthesis during inflammatory processes.	[204]
Ability to inhibit proinflammatory pathways such as those focused on the action of 5-lipoxygenase, which would otherwise lead to the possible excessive biosynthesis of leukotriene mediators of inflammation and phospholipase A2, which generates arachidonic acid and, in turn, favors the biosynthesis of inflammatory prostaglandins.	[205]
Inhibition of multiple cellular enzymes such as tyrosine kinase (TK) including growth factor receptor EGFR, calcium-phospho-lipid-dependent protein kinase (PKC), and ornithine decarboxylase (ODC), which produces polyamines known to be involved in cell proliferation and phosphoinositide kinases PI3K and PI4P-5K, involved in the proliferative responses triggered by the mitogenic pathways of signal transduction. For these last two properties, quercetin has been extensively studied in oncology, in particular with reference to the mechanisms of cell proliferation and carcinogenesis.	[206,207]
Mimics aromatase inhibitors.	[208]
Antiplatelet and cardioprotective action that limits its use in the case of concomitant intake by the patient of anticoagulant drugs such as dicoumarols.	[209]
Neuroprotective and neurotrophic action as an adjuvant therapy in the case of neurodegenerative diseases and the prevention of the same in subjects with increased susceptibility.	[210]
